# Neoadjuvant versus adjuvant chemotherapy in upper tract urothelial carcinoma: A nationwide cohort study

**DOI:** 10.1186/s12894-022-01112-6

**Published:** 2022-11-09

**Authors:** Jong Hyun Tae, Moon Soo Ha, Byung Hoon Chi, In Ho Chang, Tae-Hyoung Kim, Soon Chul Myung, Tuan Thanh Nguyen, Myoungsuk Kim, Kyung-Eun Lee, Yuwon Kim, Hyun-ki Woo, Dae-Sung Kyoung, Hasung Kim, Se Young Choi

**Affiliations:** 1 Department of Urology, Chung-Ang University Hospital, Chung-Ang University College of Medicine, 102, Heukseok-ro, Dongjak-gu, 06973 Seoul, Republic of Korea; 2 Department of Urology, Hyundae General Hospital, Chung-Ang University College of Medicine, Gyeonggi-do, Republic of Korea; 3 Department of Urology, Cho Ray Hospital, University of Medicine and Pharmacy at Ho Chi, Minh City, Vietnam; 4 Data Science Team, Evidnet. Co., Ltd, Seongnam, Gyeonggi-do, Republic of Korea; 5 Data Science Team, Hanmi Pharm. Co., Ltd, Songpa-gu, Seoul, Republic of Korea

**Keywords:** Drug therapy, Ureteral neoplasms, Survival, Chemotherapy, Population

## Abstract

**Purpose:**

This study aimed to evaluate the trend of adjuvant chemotherapy (AC) and neoadjuvant chemotherapy (NAC) in patients who underwent radical nephroureterectomy with bladder cuff excision (NUx) for upper tract urothelial carcinoma (UTUC) to compare the perioperative outcomes and overall survival (OS) between AC and NAC using nationwide population-based data.

**Materials and methods:**

We collected data on patients diagnosed with UTUC and treated with NUx between 2004 and 2016 using the National Health Insurance Service database, and evaluated the overall treatment trends. The AC and NAC groups were propensity score-matched. Cox proportional hazard and Kaplan-Meier analyses were used to assess survival.

**Results:**

Of the 8,705 enrolled patients, 6,627 underwent NUx only, 94 underwent NAC, and 1,984 underwent AC. The rate of NUx without perioperative chemotherapy increased from 70.8 to 78.2% (R^2^ = 0.632; p < 0.001). The rates of dialysis (p = 0.398), TUR-BT (p = 1.000), and radiotherapy (p = 0.497) after NUx were similar. In the Kaplan-Meier curve, the NAC and AC groups showed no significant difference (p = 0.480). In multivariate analysis, treatment with AC or NAC was not associated with OS (hazard ratio 0.83, 95% confidence interval 0.49–1.40, p = 0.477).

**Conclusion:**

The use of NUx without perioperative chemotherapy has tended to increase in South Korea. Dialysis, TUR-BT, and radiotherapy rates after NUx were similar between the NAC and AC groups. There was no significant difference in OS between the NAC and AC groups. Proper perioperative chemotherapy according to patient and tumor conditions should be determined by obtaining more evidence of UTUC.

## Introduction

Upper tract urothelial carcinoma (UTUC) is a relatively rare disease, accounting for less than 10% of all urothelial cancers [1]. However, the prevalence of UTUC has increased slightly due to the development of diagnostic tools [2]. Recently overall incidence of UTUC decreased because of decreasing tobacco or occupational exposure [3]. The stages were various according to reports. In one study, approximately 60% of UTUCs were diagnosed in the invasive stage [4]; however, in the other, about 72% of UTUCs have been diagnosed as T2 or lower [5]. The first option for the treatment of non-metastatic UTUC is radical nephroureterectomy with bladder cuff excision (NUx). Even with proper treatment, invasive UTUC shows poor prognosis. The 5-year survival rate of the T3 stage is approximately 40% and the median survival of the T4 stage is approximately 6 months [6]. Therefore, additional treatment is needed to improve survival outcomes.

Perioperative chemotherapy is an option; however, the level of evidence is low. Because the prevalence of UTUC is low, most previous studies were retrospective and had small sample sizes. Recently, a randomized controlled trial, the POUT trial, investigated adjuvant chemotherapy (AC) in UTUC: 261 patients with pT2–T4 pN0–N3 M0 or pTany N1–3 M0 were enrolled, and the AC regimen was cisplatin or carboplatin and gemcitabine [7]. During the median follow-up of 30 months, patients treated with AC after NUx showed improved disease-free survival compared to only NUx [7]. These results have changed the landscape of the management of UTUC [8]. However, to date, no trial has directly compared AC and neoadjuvant chemotherapy (NAC). Additional evidence of the efficacy of NAC for UTUC is needed.

In 2018, in South Korea, renal pelvis cancer was diagnosed in 689 patients (0.3% of all cancers), while ureter cancer was diagnosed in 686 patients (0.3% of all cancers) [9]. The incidence of UTUC was relatively low compared to that of bladder cancer (4,577 patients; 1.9% of all cancers in 2018) [9]. However, the incidence rate of UTUC (approximately 30% that of bladder cancer) was higher than that previously reported (approximately 10% that of bladder cancer in other references) [1, 9]. Therefore, effective treatment of UTUC could be important in the treatment setting of urothelial cancer in South Korea.

In this study, we evaluated the trend of AC and NAC in patients with NUx and compared the perioperative outcomes and overall survival (OS) between the AC and NAC groups using nationwide population-based data.

## Materials and methods

### Database

We collected data from the National Health Insurance Service (NHIS) database. The NHIS is a universal health-coverage system in South Korea. More than 97% of Korean citizens (over 50 million individuals) are enrolled in the NHIS. This study was approved by the Institutional Review Board at the Chung-Ang University Hospital.

### Study design

**Fig. 1:**
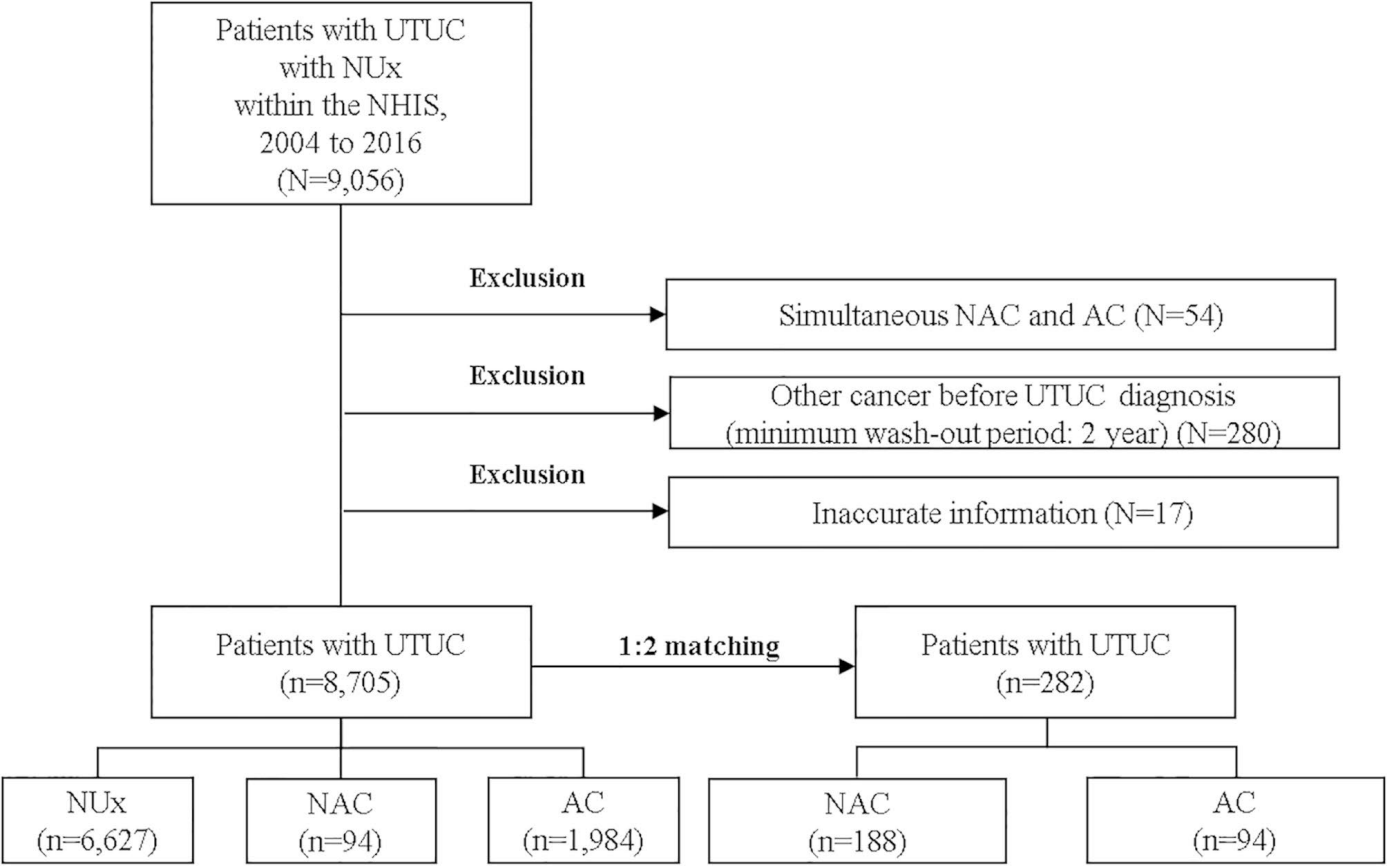
Flow chart of the study design UTUC, upper tract urothelial carcinoma; NHIS, National Health Insurance Service; AC, adjuvant chemotherapy; NAC, neoadjuvant chemotherapy; NUx, nephroureterectomy

The period of the original cohort was between 2002 and 2018, and we chose a 2-year washout period (2002–2003) and a 2-year follow-up period (2017–2018). All patients were classified under the UTUC diagnostic codes (C65 and C66), and were treated with NUx (R3432) between 2004 and 2016. The International Classification of Diseases (ICD) codes were used to confirm the diagnosis. We excluded all patients who experienced other cancer diagnoses within two years, underwent simultaneous NAC and AC, or had inaccurate information. The NAC group was defined as those using chemotherapy during the 3 months before NUx, and the AC group was defined as those using chemotherapy during the 3 months after NUx. Chemotherapy regimens included gemcitabine and cisplatin, or methotrexate, vinblastine, doxorubicin, and cisplatin (MVAC). A flowchart of the study design is shown in Fig. 1.

### Variables

Patient variables included age, year of diagnosis, sex, and medical diagnostic history. Comorbidity status was calculated from the Charlson comorbidity index (CCI) [10]. We also collected data including periods from NUx to NAC or AC, granulocyte colony-stimulating factor (G-CSF) usage, hospitalization duration at NUx, transfusion at NUx, readmission after NUx not related to chemotherapy, and dialysis after NUx.

### Statistical analyses

Clinical trends are expressed as mean ± standard deviation or numbers with percentages. Differences between groups were compared using Student’s t-test and ANOVA for continuous variables, and chi-square test for categorical variables. Linear regression was performed to estimate the treatment trends. OS was calculated from the date of diagnosis to the date of the last follow-up or death, and was estimated using the Kaplan-Meier method with the log-rank test. Propensity score matching (PSM) was performed based on age, year of diagnosis, sex, and CCI. We performed 1:2 nearest matching. A Cox proportional hazards model was used for multivariate analysis. All statistical analyses were performed using SAS v.7.0 (SAS Institute Inc., Cary, NC, USA) and R software, version 4.0.1 (R Foundation for Statistical Computing, Vienna, Austria). Statistical significance was set at p < 0.05.

## Results

The final cohort consisted of 8,705 patients (Table 1), comprising 6,627 patients who underwent NUx only (NUx group), 94 patients who underwent NAC and NUx (NAC group), and 1,984 patients who underwent AC and NUx (AC group). There was no significant difference in age between the NUx, AC, and NAC groups (67.9 ± 10.4 [median 70.0 interquartile range 61.0–76.0], 64.2 ± 9.3 [median 65.0 interquartile range 58.0–71.0], and 65.2 ± 9.4 [median 67.0 interquartile range 60.0–72.0], respectively, p = 0.294). Before PSM, the rates of NAC were higher in the 2010–2016 and CCI < 4 groups (p < 0.001 and P = 0.010, respectively). After PSM, the variables age, sex, diagnostic year, and CCI were well matched between groups (all standardized mean differences < 0.1).

**Table 1:** Baseline characteristics of the study population

	** Before match**					** After match**			
	**NUx**	**AC**	**NAC**	**p-value**		**AC**	**NAC**	**p-value**	**SMD**
	**(n = 6627)**	**(n = 1984)**	**(n = 94)**	**(AC vs. NAC)**		**(n = 188)**	**(n = 94)**		
Age	67.9 ± 10.4	64.2 ± 9.3	65.2 ± 9.4	0.294		65.0 ± 8.9	65.2 ± 9.4	0.875	0.020
	70.0 [61.0;76.0]	65.0 [58.0;71.0]	67.0 [60.0;72.0]			66.0 [59.5;72.0]	67.0 [60.0;72.0]		
Age group				0.213				1.000	< 0.001
- <65	2236 (33.7%)	922 (46.5%)	37 (39.4%)			74 (39.4%)	37 (39.4%)		
- ≥65	4391 (66.3%)	1062 (53.5%)	57 (60.6%)			114 (60.6%)	57 (60.6%)		
Sex				0.333				1.000	< 0.001
- Male	4643 (70.1%)	1452 (73.2%)	64 (68.1%)			128 (68.1%)	64 (68.1%)		
- Female	1984 (29.9%)	532 (26.8%)	30 (31.9%)			60 (31.9%)	30 (31.9%)		
Diagnostic year				< 0.001				0.722	0.065
− 2004–2009	2062 (31.1%)	779 (39.3%)	19 (20.2%)			43 (22.9%)	19 (20.2%)		
− 2010–2016	4565 (68.9%)	1205 (60.7%)	75 (79.8%)			145 (77.1%)	75 (79.8%)		
CCI				0.010				1.000	< 0.001
- <4	4285 (64.7%)	1455 (73.3%)	57 (60.6%)			114 (60.6%)	57 (60.6%)		
- ≥4	2342 (35.3%)	529 (26.7%)	37 (39.4%)			74 (39.4%)	37 (39.4%)		

Data are presented as n (%), mean ± standard deviation, or median [interquartile range]

Abbreviations: NUx, Nephroureterectomy; AC, Adjuvant chemotherapy; NAC, Neoadjuvant chemotherapy; SMD, Standardized mean difference; CCI, Charlson Comorbidity Index

**Fig. 2:**
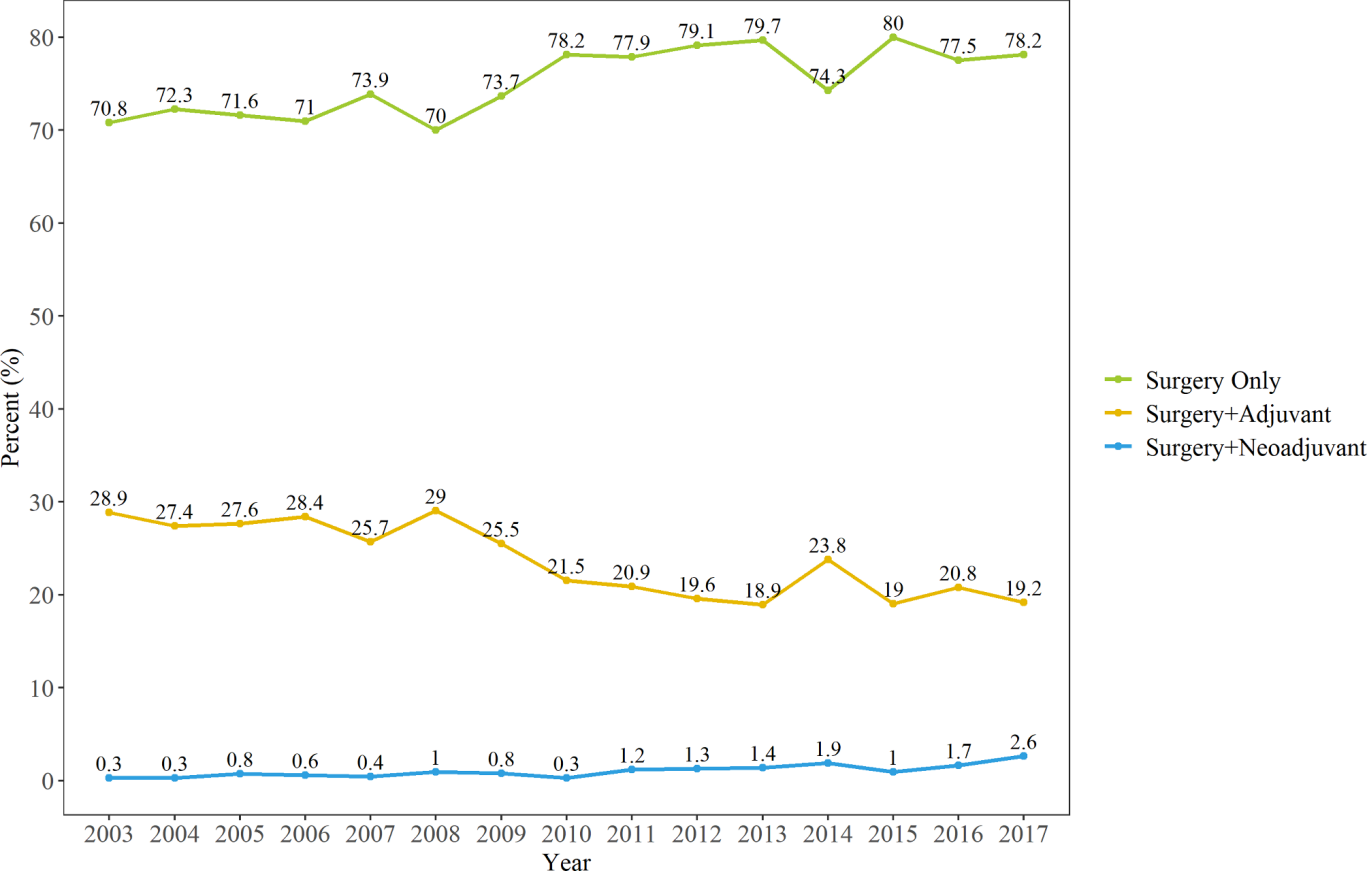
Application of upper tract urothelial carcinoma treatment strategies

Figure 2 shows the trends of UTUC treatment utilization. The rates of patients who underwent NUx and NAC increased from 2003 to 2017 (NUx: 70.8–78.2%, R^2^ = 0.632, p < 0.001; NAC: 0.3–2.6%, R^2^ = 0.691, p < 0.001). Concomitantly, the rate of patient who udnerwent AC decreased from 28.9 to 19.2% (R^2^ = 0.746; p < 0.001). The estimated annual percentage change was 12.29% (95% confidence interval [CI]: 1.4–24.34%), estimates in NAC and was 10.23% (95% CI: 0.76–20.59%) estimates in AC.

The clinical trends are presented in Table 2. The median period between NUx and the first day of NAC was 4.0 months and the median period from NUx to the first day of AC was 1.1 months. There was no difference in hospitalization duration at NUx (p = 0.148) or 3-month readmission rates after NUx (p = 0.502). The rate of transfusion at NUx was higher in the NUx group than in the AC group (62.8% vs. 35.1%, p < 0.001); however, the volume of transfusion was statistically similar (p = 0.678). The G-CSF usage rate was higher in the AG group than in the NAC group (33.0% vs. 16.0%, p = 0.004); however, there was no difference in the number of G-CSF usages during chemotherapy, for total period or within 3-months. The dialysis rate after NUx did not differ between the groups. The rates of preoperative biopsy or transurethral resection of bladder tumor (TUR-BT) were higher in the NAC group (p = 0.006 and p < 0.001, respectively). The rate of TUR-BT after NUx was similar (p = 1.000), and the mean periods from NUx to TUR-BT were 15.6 ± 13.1 months in the AC group and 15.1 ± 16.8 months in the NAC group (p = 0.910). The radiotherapy rate after NUx was similar (P = 0.497).

**Table 2:** Clinical trends after treatment

	** Before matching**						** After matching**				
	**AC**		**NAC**		**p-value**		**AC** **(n = 188)**		**NAC** **(n = 94)**		**p-value**
	**(n = 1984)**		**(n = 94)**								
	Mean ± SD	Median [Q1; Q3]	Mean ± SD	Median [Q1; Q3]			Mean ± SD	Median [Q1; Q3]	Mean ± SD	Median [Q1; Q3]	
Period from NUx to ST (months)	1.2 ± 0.5	1.1 [ 0.9; 1.4]	4.8 ± 3.0	4.0 [ 2.6; 6.1]	< 0.001		1.2 ± 0.5	1.1 [ 0.9; 1.4]	4.8 ± 3.0	4.0 [ 2.6; 6.1]	< 0.001
Hospitalization duration at NUx (days)	13.2 ± 6.3	11.0 [10.0;15.0]	14.1 ± 8.6	12.0 [10.0;16.0]	0.323		12.7 ± 6.0	11.0 [ 9.0;15.0]	14.1 ± 8.6	12.0 [10.0;16.0]	0.148
3-months readmission rate after NUx (%)					0.063						0.502
- No	1451 (73.1%)		60 (63.8%)				129 (68.6%)		60 (63.8%)		
- Yes	533 (26.9%)		34 (36.2%)				59 (31.4%)		34 (36.2%)		
Transfusion at NUx (%)					< 0.001						< 0.001
- No	1206 (60.8%)		35 (37.2%)				122 (64.9%)		35 (37.2%)		
- Yes	778 (39.2%)		59 (62.8%)				66 (35.1%)		59 (62.8%)		
Volume of transfusion at NUx (packs)	3.3 ± 2.6	2.0 [ 2.0; 4.0]	3.2 ± 2.0	2.0 [ 2.0; 4.0]	0.872		3.6 ± 3.0	3.0 [ 2.0; 4.0]	3.2 ± 2.0	2.0 [ 2.0; 4.0]	0.678
G-CSF usage rate (%)					< 0.001						0.004
- No	1272 (64.1%)		79 (84.0%)				126 (67.0%)		79 (84.0%)		
- Yes	712 (35.9%)		15 (16.0%)				62 (33.0%)		15 (16.0%)		
The number of G-CSF usage, during ST	2.7 ± 3.0	2.0 [ 1.0; 3.0]	2.1 ± 1.9	1.0 [ 1.0; 2.5]	0.445		2.4 ± 2.0	2.0 [ 1.0; 3.0]	2.1 ± 1.9	1.0 [ 1.0; 2.5]	0.603
The number of G-CSF usage, during total period	2.8 ± 3.1	2.0 [ 1.0; 3.0]	2.3 ± 1.8	2.0 [ 1.0; 3.0]			2.6 ± 2.2	2.0 [ 1.0; 3.0]	2.3 ± 1.8	2.0 [ 1.0; 3.0]	0.558
The rate of G-CSF usage within 3-months (%)					0.182						0.147
- No	1659 (83.6%)		84 (89.4%)				154 (81.9%)		84 (89.4%)		
- Yes	325 (16.4%)		10 (10.6%)				34 (18.1%)		10 (10.6%)		
Dialysis after NUx					0.278						0.398
- No	1895 (95.5%)		87 (92.6%)				180 (95.7%)		87 (92.6%)		
- Yes	89 (4.5%)		7 (7.4%)				8 (4.3%)		7 (7.4%)		
Preoperative URS biopsy (%)					< 0.001						0.006
- No	1658 (83.6%)		65 (69.1%)				158 (84.0%)		65 (69.1%)		
- Yes	326 (16.4%)		29 (30.9%)				30 (16.0%)		29 (30.9%)		
Preoperative TUR-BT (%)					< 0.001						< 0.001
- No	1759 (88.7%)		64 (68.1%)				166 (88.3%)		64 (68.1%)		
- Yes	225 (11.3%)		30 (31.9%)				22 (11.7%)		30 (31.9%)		
TUR-BT after NUx (%)					0.866						1.000
- No	1472 (74.2%)		71 (75.5%)				141 (75.0%)		71 (75.5%)		
- Yes	512 (25.8%)		23 (24.5%)				47 (25.0%)		23 (24.5%)		
Period from NUx to TUR-BT (months)	17.4 ± 16.4	12.2 [ 8.4;21.0]	15.1 ± 16.8	10.0 [ 3.9;15.0]	0.523		15.6 ± 13.1	12.2 [ 7.8;21.6]	15.1 ± 16.8	10.0 [ 3.9;15.0]	0.910
The rate of radiotherapy (%)					0.860						0.497
- No	1750 (88.2%)		84 (89.4%)				174 (92.6%)		84 (89.4%)		
- Yes	234 (11.8%)		10 (10.6%)				14 (7.4%)		10 (10.6%)		

Abbreviations: AC, Adjuvant chemotherapy; NAC, Neoadjuvant chemotherapy; SD, Standard Deviation; NUx, Nephroureterectomy; ST, systemic therapy;

G-CSF, Granulocyte Colony Stimulating Factor; URS, ureterorenoscopy; TUR-BT, Transurethral resection of bladder tumor

**Fig. 3:**
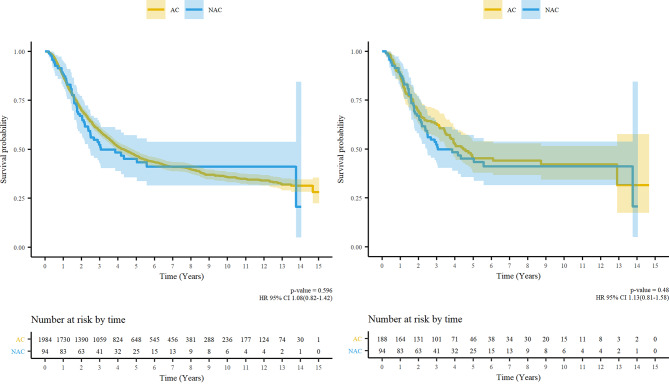
Kaplan-Meier Survival Curves and Log-Rank Tests for overall survival (A) Before matching, and (B) After matching AC, Adjuvant chemotherapy; NAC, Neoadjuvant chemotherapy; HR, hazard ratio; CI, confidence interval

**Fig. 4:**
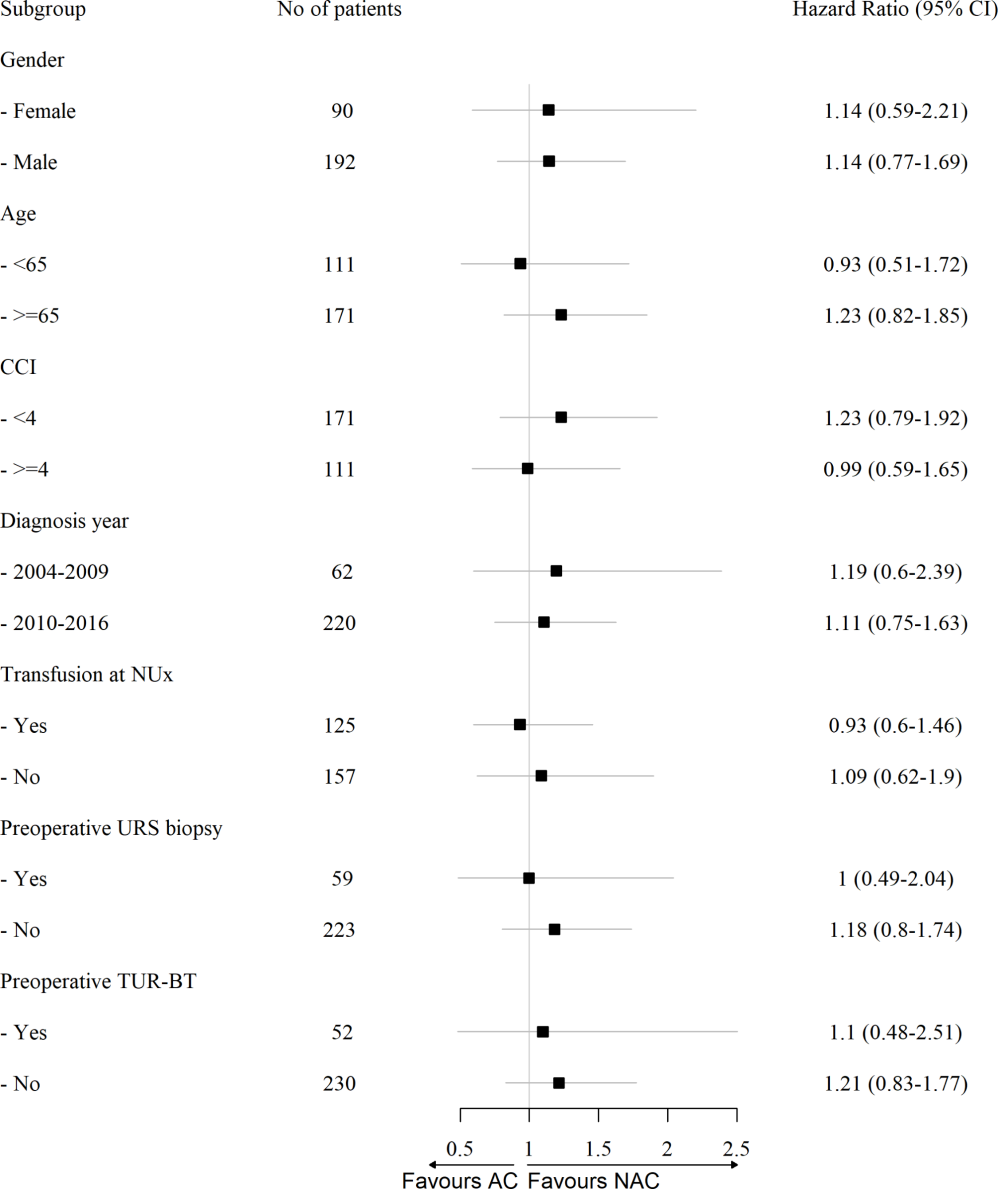
Forest plot classified by chemotherapy regimen CCI, Charlson comorbidity index; NUx, nephroureterectomy; URS, ureterorenoscopy; TUR-BT, transurethral resection of bladder tumor

Before PSM, the 5-year OS rates were 49.5% and 47.9% in the AC and NAC groups, respectively. The NAC group showed a similar OS to that of the AC group based on the Kaplan– Meier curve (p = 0.596, Fig. 3 A). After PSM, the OS in the NAC group did not differ from that in the AC group (P = 0.480, Fig. 3B). In multivariate analysis (Table 3), treatment with AC or NAC was not associated with OS (before PSM: hazard ratio [HR] 1.05, 95% CI 0.79–1.39, p = 0.733; after PSM: HR 0.83, 95% CI 0.49–1.40, p = 0.477). Subgroup analysis did not show any significance (Fig. 4).

**Table 3:** Hazard ratio and 95% confidence interval of overall survival according to treatment and incidence rate

	** Before matching**							** After matching**			
**Variable**		**Incidence**	**Univariate**	**p-value**	**Multivariate**	**p-value**		**Univariate**	**p-value**	**Multivariate**	**p-value**
Treatment											
- AC		126.44	1		1			1		1	
- NAC		147.643	1.08(0.82–1.42)	0.596	1.05(0.79–1.39)	0.733		1.13(0.81–1.58)	0.480	0.83(0.49–1.40)	0.477
Age group											
- <65		98.102	1		1			1		1	
- ≥65		158.822	1.5(1.34–1.69)	< 0.001	1.49(1.32–1.68)	< 0.001		1.46(1.03–2.06)	0.032	1.3(0.91–1.86)	0.146
Gender											
- Female		115.667	1		1			1		1	
- Male		131.678	1.12(0.98–1.28)	0.085	1.17(1.03–1.34)	0.019		1.58(1.09–2.3)	0.015	1.59(1.09–2.33)	0.016
CCI											
- <4		118.348	1		1			1		1	
- ≥4		158.249	1.19(1.04–1.35)	0.009	1.1(0.96–1.26)	0.157		1.32(0.95–1.84)	0.092	1.3(0.92–1.82)	0.133
Chemotherapy duration											
- <2 months		119.486	1		1			1		1	
- ≥2 months		159.953	1.31(0.91–1.87)	0.142	1.26(0.91–1.75)	0.167		1.3(0.93–1.81)	0.130	1.51(0.9–2.54)	0.119
Diagnosis year											
− 2004–2009		108.42	1		1			1		1	
− 2010–2016		147.96	1.02(0.9–1.15)	0.773	0.98(0.86–1.1)	0.686		1.1(0.74–1.62)	0.647	0.95(0.63–1.42)	0.803

Abbreviations: AC, Adjuvant chemotherapy; NAC, Neoadjuvant chemotherapy; CCI, Charlson Comorbidity Index

Multivariate analyses about Treatment were performed by Age, Gender, CCI, chemotherapy duration and Diagnosis year

## Discussion

The POUT trial provided a higher level of evidence of the benefit (disease-free survival: HR 0.45, 95% CI 0.30–0.68) of AC for UTUC patients with pathologic T2 or higher [7]. However, there has been no randomized controlled trial investigating the efficacy of NAC. In a relatively recent phase 2 trial, NAC in high-grade UTUC showed complete pathological response in 13.8% of cases [11]. In this study, we assessed the utility trend of NAC and AC in UTUC patients in South Korea using nationwide population-based data. The utility rate of perioperative chemotherapy has decreased from approximately 29% in 2003 to 22% in 2017 in South Korea. In the Netherlands, only 3.8% of patients within the Surveillance, Epidemiology, and End Results database (2004–2014) received perioperative chemotherapy from 2013 to 2017 [12], while 37.8% of patients who had UTUC of stage T3 or higher received perioperative chemotherapy. The use of perioperative chemotherapy in UTUC shows different trends across different countries and time periods. There is no clear consensus between guidelines due to the lack of evidence and the rarity of UTUC [8, 13].

Perioperative chemotherapy consists of AC and NAC. AC can be administered at an accurate pathological stage. The preoperative clinical stage is generally evaluated using imaging tools such as computed tomography or magnetic resonance imaging; however, these methods have limitations in predicting the accurate pathologic stage [14]. With accurate pathologic staging, the possibility of overtreatment with AC can be reduced. In the subgroup analysis of the POUT trial, patients classified as stage T3 or higher showed a greater increase in disease-free survival after AC [7]. However, a decline in renal function after NUx could reduce the number of patients who should receive AC. In one prior study, the estimated glomerular filtration rate (GFR) decreased by ~ 24% after NUx and 30% of patients showed decreases in GFR of under 60 ml/min, which would make them ineligible for gemcitabine and cisplatin chemotherapy [15]. In the POUT trial, subjects with impaired renal function (GFR 30–50 ml/min) were permitted to undergo the regimen of gemcitabine and carboplatin [7]. However, the subgroup that received gemcitabine and carboplatin did not show significant benefit [7]. Of course the subgroup was underpowered to conclude, but the proper regimen and enroll condition should be defined.

In a meta-analysis, patients who underwent NAC showed a significantly better pathologic complete response rate, pathologic downstaging rate, OS, and cancer-specific survival than patients who underwent NUx alone [16]. The advantage of NAC is that the relatively higher GFR before NUx allows more patients to undergo the optimal chemotherapy regimen. In the VESPER trial, which investigated patients who received either gemcitabine and cisplatin or MVAC as NAC in muscle-invasive bladder cancer, poorer GFR (50–59 ml/min) showed a lower possibility of organ-confined disease than normal GFR (≥ 90 ml/min) [17]. Cisplatin could induce severe nephrotoxicity, which is related to dose limitation and treatment discontinuation [18]. Cisplatin can injure the proximal tubule and vessel by inflammation and oxidative stress [19]. The cumulative dose of cisplatin could be a risk factor for renal toxicity [20]. Despite the relatively favorable GFR at NAC, NAC has limitations in taking the possibility of cure by earlier surgery in patients who are non-responders to chemotherapy.

Previously, we reported that NAC showed a better OS than AC for bladder cancer using nationwide population-based data [21]. In this study, we found no difference in OS between NAC and AC for UTUC. UTUC and bladder share similar risk factors and grading systems [22]. However more than 50% of UTUC patients are diagnosed in the advanced stages of the disease, whereas 20% of bladder cancer patients are diagnosed in the advanced stages [23]. One reason for this could be that the thick bladder muscle acts as a protective barrier against the tumor, which is absent in the ureter. Another reason may be the different genetic and epigenetic characteristics of UTUC and bladder cancer [24]. More clinical evidence is needed to optimize the management of UTUC patients to improve survival outcomes of patients with UTUC.

Even though we collected almost complete enumeration data in South Korea to cover the cost of cancer treatment, the small sample size is a limitation. However, most prior studies on UTUC also had small sample sizes because of the rarity of UTUC. In particular, the scarce evidence regarding NAC leads to hesitation amongst clinicians on its use. Therefore, further studies on NAC are needed. The retrospective nature of the study possibly introduced selection bias. In addition, there is currently no information regarding tumor stage and performance status. However, we tried to exclude the chemotherapy for metastatic UTUC. NHIS service covered pathologic T3 or more or pathologic lymph node positive as AC. We hypothesized perioperative chemotherapy used for locally advanced stage, not metastatic stage. We could not also detect cancer-specific mortality. A randomized controlled trial (URANUS) on NAC, AC, and NUx is ongoing, which will hopefully suggest the proper treatment for UTUC [25].

## Conclusion

The use of NUx without perioperative chemotherapy to treat UTUC has increased in South Korea. In this study, we found that the rates of dialysis, TUR-BT, and radiotherapy after NUx were similar between the NAC and AC groups. There was no significant difference in OS between the NAC and AC groups. Proper perioperative chemotherapy according to UTUC patient and tumor conditions should be better determined after accumulating more evidence on this cancer.

## Data Availability

The data that support the findings of this study are available from the National Health Insurance Service but restrictions apply to the availability of these data, which were used under license for the current study, and so are not publicly available. Data are however available from the authors upon reasonable request and with permission of the National Health Insurance Service.

## Abbreviations

UTUC: Upper tract urothelial carcinoma.
NUx: Radical nephroureterectomy with bladder cuff excision.
AC: Adjuvant chemotherapy.
NAC: Neoadjuvant chemotherapy.
OS: Overall survival.
NHIS: National Health Insurance Service.
ICD: International Classification of Diseases.
MVAC: Methotrexate, vinblastine, doxorubicin, cisplatin.
CCI: Charlson comorbidity index.
G-CSF: Granulocyte colony-stimulating factor.
PSM: Propensity score matching.
TUR-BT: Transurethral resection of bladder tumor.
HR: Hazard ratio.
CI: Confidence interval.
GFR: Glomerular filtration rate.
